# Forgetting of long-term memory requires activation of NMDA receptors, L-type voltage-dependent Ca^2+^ channels, and calcineurin

**DOI:** 10.1038/srep22771

**Published:** 2016-03-07

**Authors:** Ricardo Marcelo Sachser, Fabiana Santana, Ana Paula Crestani, Paula Lunardi, Lizeth Katherine Pedraza, Jorge Alberto Quillfeldt, Oliver Hardt, Lucas de Oliveira Alvares

**Affiliations:** 1 Neurobiology of Memory Lab, Biophysics Department, Bioscience Institute, Federal University of Rio Grande do Sul, Porto Alegre, Rio Grande do Sul, Brazil; 2 Psychobiology and Neurocomputation Lab, Biophysics Department, Bioscience Institute, Federal University of Rio Grande do Sul, Porto Alegre, Rio Grande do Sul, Brazil; 3 Graduate Program in Neuroscience, Federal University of Rio Grande do Sul, Porto Alegre, Rio Grande do Sul, Brazil; 4 Centre for Cognitive and Neural Systems, University of Edinburgh, Edinburgh, Scotland; 5 Laboratório de Neurobiologia da Memória, Departamento de Biofísica, Instituto de Biociências, Universidade Federal do Rio Grande do Sul (UFRGS), Av. Bento Gonçalves 9500, Prédio 43422, Sala 216A, CEP 91.501-970, Porto Alegre, Rio Grande do Sul, Brasil

## Abstract

In the past decades, the cellular and molecular mechanisms underlying memory consolidation, reconsolidation, and extinction have been well characterized. However, the neurobiological underpinnings of forgetting processes remain to be elucidated. Here we used behavioral, pharmacological and electrophysiological approaches to explore mechanisms controlling forgetting. We found that post-acquisition chronic inhibition of the N-methyl-D-aspartate receptor (NMDAR), L-type voltage-dependent Ca^2+^ channel (LVDCC), and protein phosphatase calcineurin (CaN), maintains long-term object location memory that otherwise would have been forgotten. We further show that NMDAR activation is necessary to induce forgetting of object recognition memory. Studying the role of NMDAR activation in the decay of the early phase of long-term potentiation (E-LTP) in the hippocampus, we found that ifenprodil infused 30 min after LTP induction *in vivo* blocks the decay of CA1-evoked postsynaptic plasticity, suggesting that GluN2B-containing NMDARs activation are critical to promote LTP decay. Taken together, these findings indicate that a well-regulated forgetting process, initiated by Ca^2+^ influx through LVDCCs and GluN2B-NMDARs followed by CaN activation, controls the maintenance of hippocampal LTP and long-term memories over time.

The ability to retain long-term records of daily experiences may seem as the most critical feature of memory, yet most memories will be forgotten within a couple of days^1^. Several theoretical positions have been developed to account for memory loss over time, which has been viewed (a) as a progressive degradation with the passage of time, (b) as the result of new learning that interferes with memory storage or expression, or (c) as the result of some form of retrieval error^2^,^3^,^4^,^5^.

Although the cellular and molecular mechanisms underpinning memory consolidation, reconsolidation, and extinction are well known, the biological nature of long-term memory (LTM) forgetting remains poorly understood. The few studies investigating forgetting have described a role for NDMAR activation in time-dependent memory loss. Villarreal *et al.*^6^ showed in rats that after LTP induction *in vivo*, systemic injections of the NMDAR antagonist CPP sustained perforant-path LTP^6^. Moreover, systemic injections of CPP also maintained spatial (radial arm maze) memory, suggesting that NMDAR activation is robustly involved in the decay of both the late phase of LTP (L-LTP) and spatial LTM^6^. Similar results were obtained with daily intrahippocampal injections of the NMDAR antagonist D-AP5 in Morris water maze^7^.

The influx of Ca^2+^ through NMDARs activates several proteins that regulate LTP and long-term depression (LTD). In fact, LTD in both the amygdala^8^ and the hippocampus^9^, are dependent on Ca^2+^ influx from GluN2B-containing NMDAR. Interestingly, fear extinction requires GluN2B-mediated signaling^8^, and strong emotional memories that are not susceptible to disruption express lower GluN2B-NMDAR levels^10^. In light of these findings, Hardt *et al.*^4^,^5^ suggested that the metaplastic ratio of postsynaptic GluN2B to GluN2A-containing NMDARs may present a central factor regulating the persistence of L-LTP and LTM^4^,^5^. According to this model, a relatively higher expression of GluN2B-NMDARs will increase the probability of certain forms of postsynaptic plasticity, permitting depotentiation (or LTD), possibly through a mechanism that control trafficking of GluA2-AMPARs^4^,^5^,^11^. In addition to NMDAR activation, Ca^2+^ influx through LVDCCs may also participate in processes that promote forgetting. For example, LVDCCs critically contribute to several important molecular cascades associated with postsynaptic plasticity, such as fear memory destabilization following retrieval^12^,^13^,^14^.

The protein calcineurin (CaN) is a Ca^2+^ -dependent serine/threonine phosphatase that is robustly implicated in the modulation of learning and memory^15^,^16^,^17^,^18^,^19^. It is well known that phosphorylation of several protein kinases is responsible for enhanced LTP by increasing the number of AMPARs in the postsynaptic density^20^, while the activity-dependent dephosphorylation of AMPAR is regulated by CaN, which is implicated in the depontentiation rate of CA1-evoked plasticity, specifically through bidirectional interactions with the transmembrane AMPAR regulatory protein stargazin^21^. Additionally, Jouvenceau *et al.*^17^ demonstrated that CaN, but not other phosphatases such as PP1 or PP2A, positively modulates the depotentiation of LTP, since the CaN inhibitor FK506 impairs this process in hippocampal-treated slices, but had no effect on LTD-dependent pathway^17^. The same study also showed that reversible genetic inhibition of calcineurin blocked depotentiation^17^.

In the present study we therefore used behavioral and pharmacological approaches to explore mechanisms that regulate the persistence of both long-term object location (OL) and object recognition (OR) memories in rats. We show that these processes require activation of LVDCCs and NMDARs. Furthermore, we demonstrate that after hippocampal LTP induction *in vivo*, GluN2B-NMDARs are critical to sustain E-LTP in a nondecaying state. We also show that daily systemic inhibition of CaN maintained OL memory, indicating that CaN activation participates in processes that determine whether a memory will persist or be forgotten. Taken together, these findings suggest that, after the establishment of LTM, the activation of NMDARs and LVDCCs leads to increased Ca^2+^ levels, which activate CaN, thus promoting forgetting.

## Material and Methods

### Animals

Male Wistar rats (age 2–3 months, weight 290–350 g) from our breeding colony were used. Animals were housed in plastic cages, four to five per cage, with water and food available *ad libitum* under a 12/12 h light/dark cycle (lights on at 7 a.m.) in a constant temperature of 24 ± 1 °C. All experiments were performed in accordance to the national animal care legislation and guidelines (Brazilian Law 11794/2008), and approved by the Ethics Committee of the Federal University of Rio Grande do Sul.

### Behavioral procedures

For object location (OL) and object recognition (OR) tasks we used a black square arena (60 × 60 × 60 cm) made of wood with distinct visual cues in the walls. The general protocol for both paradigms consisted of three distinct phases: (1) 4 consecutive days of 10-min exposure for habituation in the absence of objects, (2) 2 days of 10-min training session with two identical objects positioned in opposite corners (15 cm distant of the walls), and (3) 5-min test session for memory retention. Test sessions were performed 1, 3, 5, 7 or 10 days after the last day of training, depending of the experiment. For OL retention test, one of the identical objects was displaced into a different position in the arena. For OR test, a novel object was switched without modifying its original position from the training. The objects consisted of rectangular and circular metal cans. Behavioral experiments were conducted only at daytime, being performed in a silent illuminated room (constant temperature of 26 ± 1 °C). All components of our apparatus were thoroughly cleaned with 70% ethanol solution between each trial to make sure no olfactory cues were present. A video camera installed above the arena was used to record the experiments, which was quantified by a blind observer to drug treatments.

### Drugs

In the behavioral procedures, the first dose of all drugs was initially injected i.p. or s.c. 6 h after the last day of training session. Two NMDAR antagonists were used, as follows: memantine hydrochloride (Tocris) (10 or 20 mg/kg) dissolved in sterile isotonic saline with 8% DMSO, and MK801 (Sigma) (0.1 mg/kg) dissolved in sterile isotonic saline. Because of individual bioavailability properties of NMDAR antagonists, memantine was administered once a day, and MK801 each 12 h (because of its shorter half-life) for 1 week or 10 days, depending on the protocol. Both drugs were injected i.p. The L-type voltage-dependent calcium channels (LVDCCs) inhibitor nimodipine was purchase from Sigma-Aldrich, and dissolved in sterile isotonic saline with 8% DMSO to a concentration of 16 mg/mL. Nimodipine or its vehicle was injected s.c. once a day for 1 week. FK506 (Sigma-Aldrich) (5 mg/kg), an inhibitor of the protein phosphatase calcineurin, was dissolved in sterile isotonic saline with 20% DMSO. FK506 or its vehicle was administered i.p. once per day for 1 week. All drugs in the behavioral experiments were injected in a total volume adjusted in 1 mg/mL. In the electrophysiology, we used the selective antagonist of the GluN2B-NMDAR ifenprodil (Sigma-Aldrich) that was dissolved in a phosphate buffered saline to a concentration of 0.1 or 1 mg/mL. The doses were based on previous studies^22^,^23^,^24^. Ifenprodil or its vehicle was infused into the CA1 area of the dorsal hippocampus 30 min after LTP induction (unilateral infusion of 0.5 μL at a slow rate of 20 μL/h).

### Electrophysiology

Field excitatory postsynaptic potentials (fEPSP) were recorded for 180 min after LTP induction in the dorsal CA1 region of the hippocampus *in vivo*. Rats were anesthetized with urethane (1.5 mg/kg), carefully placed in a stereotaxic frame (Kopf), and concentric bipolar stimulating electrodes were positioned in the Schaffer collateral, as follows: −4.0 mm posterior to bregma, −3.0 mm from midline, with the dorsoventral coordinates individually adjusted to produce a maximal response, around −2.5 mm below the rat cerebral cortex. Glass micropipettes filled with 0.3 M sodium chloride was aimed into contralateral CA1 region with the same stereotaxic coordinates. The intensity (300–600 μA) was adjusted to a level at which the amplitude of the negative component could be reliably measured. Paired pulses were delivered at a rate of 0.1 Hz for a period of 20 min to establish individual baseline responses. Then, high-frequency stimulation (HFS) consisting of 10 trains of 100 Hz pulses for 200 ms with 2 s interval between trains were applied. The selective GluN2B-NMDAR antagonist ifenprodil or its vehicle was infused in the hippocampus 30 min after LTP induction, and responses were recorded for 180 min. For the statistics, we used the average of fEPSPs in the last 10 min prior the tetanic stimulus (baseline response) and two post-tetanic (*t*50–60 min and *t*170–180 min).

### Data collection and analysis

Memory retention was expressed as a percentage of the total exploration time to both objects. The exploration index was calculated as *t*_novel_ × 100/(*t*_familiar_ + *t*_novel_). The exploration was defined only when rats sniffs the object, being evaluated manually by a trained observer blind to experimental conditions. Within-group comparisons were performed using two tailed Student’s paired *t* test or repeated measure ANOVA. For between-group comparisons we used two tailed independent *t* test or one way ANOVA. SNK *post-hoc* test was used whenever necessary. Significance was set at *p* < 0.05. These analyses were performed using the version 8.0 of Statistica software, and graphs were made using GraphPad Prism version 6. A subject was excluded as a statistical outlier (lying more than 2 standard deviations from the group mean in the training session).

## Results

### When do rats start to forget

**Figure 1:**
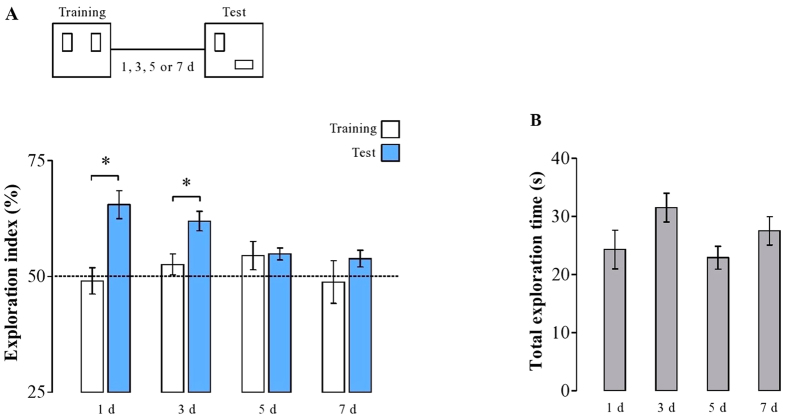
Rats maintain the object location memory in 1 and 3, but not in 5 or 7 days after learning. (**A**) Experimental design of the OL paradigm (top panel), and the exploration ratios during the training (white bars) and retention tests (blue bars) performed on days 1, 3, 5, or 7 after learning. Rats keep their memory until day 3, since they spent more time exploring the object that was switched into a new position. (**B**) The overall exploration activity in the test does not differ among all groups. Dotted line indicates similar exploration activity between both novel and familiar objects. Data expressed as mean ± SEM. ^*^p < 0.5. All other comparisons were not significant (*n* = 4 to 6 per group).

In order to determine the time course of forgetting, animals were trained in an object location (OL) task. Animals were presented with two identical copies of a junk object (metal can) located at stable positions in a familiar open field. Memory was assessed 1, 3, 5 or 7 days after training. To this end, one of the two objects was moved to a new location, and relative preference to explore this novelty was assessed as indicating long-term object location memory. The novelty preference indices for all groups and the total exploration time are shown in Fig. 1. We found that animals tested 1 or 3 days after training expressed a significant preference to the displaced object (*t*(4) = −3.29; *p* = 0.03; *t*(5) = −2.98; *p* *=* 0.03, paired *t*-test comparing exploratory preference during training and memory test). However, animals tested 5 or 7 days after training showed no preference for the displaced object (*t*(4) = 0.83; *p* = 0.44; *t*(4) = −1.58; *p* *=* 0.18, paired *t*-test), indicating that spatial memory was forgotten. The overall time spent exploring the objects was not different among the groups as revealed by one way ANOVA (*F*(3, 40) = 2.21; *p* = 0.10; Fig. 1B).

Thus, forgetting of OL memory starts sometime between 3 and 5 days after training in the present protocol.

### Role of NMDAR in forgetting: chronic systemic injections of NMDAR antagonists maintain long-term memory

**Figure 2:**
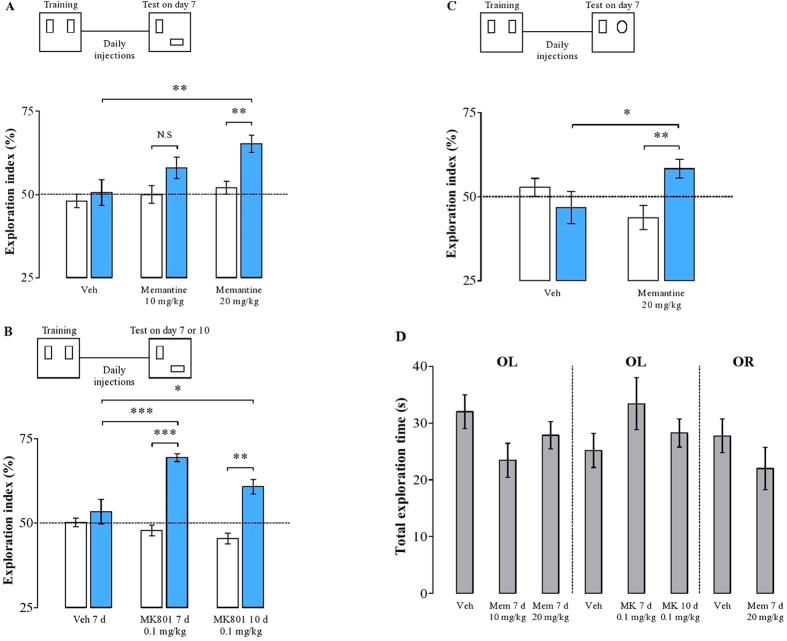
Long-term memory forgetting is regulated through NMDAR-dependent signaling. (**A**) Dose-dependent effect of the NMDAR antagonist memantine upon the test conducted 7 days after training in the OL task. Rats chronically treated with the high dose of memantine explored significantly more the displaced object compared to other groups. (**B**) The NMDAR antagonist MK801 prevent memory forgetting for either 7 or 10 days after learning in the same paradigm. (**C**) NMDAR inhibition also regulates the decay of the OR memory. White bars represent the performance in the training, and blue bars in the tests. (**D**) The overall exploration does not differ among the groups, indicating that memantine or MK801 have no effect on basal exploratory activity. Data expressed as mean ± SEM. *p < 0.5, **p < 0.01, ***p < 0.001 (*n* = 6 to 9 per group).

It has been previously shown that chronic NMDAR blockade can sustain spatial memory in the radial-arm maze^6^ as well as in the Morris water maze^7^, and that it blocks the decay of LTP in freely moving animals^6^. To explore whether this is also the case for the OL task, rats were tested in a time-point when this type of memory would normally be forgotten. After the training session, we injected systemically the NMDAR antagonist memantine (10 or 20 mg/kg) or its vehicle once a day, with the first injection 6 h after the last day of training (Fig. 2A). During the drug-free retention test performed 7 d after training, a one-way ANOVA detected a significant difference among the groups in the exploration of the displaced object (*F*(2, 17) = 5.10; *p* = 0.01). *Post hoc* comparisons revealed that the group treated with 20 mg/kg of memantine explored the displaced object significantly longer than the control group (*p* = 0.01). Additionally, both vehicle and 10 mg/kg memantine-treated groups expressed no preference for the object at the novel location compared to exploration during training (*t*(5) = −0.84; *p* = 0.43; *t*(6) = −1.81; *p* *=* 0.11, paired *t-*tests). However, the group treated with 20 mg/kg of memantine preferred exploring the displaced object (*t*(5) = −3.41; *p* = 0.01, paired *t*-test). The overall time spent exploring the objects was not different among the groups, as revealed by one-way ANOVA (*F*(2, 33) = 2.25; *p* = 0.12; Fig. 2D, left panel). Moreover, to determine whether this effect could have been caused by an early NMDAR blockade (around 6–12 h after training), we trained another set of animals and tested them 7 d later. In contrast to the previous experiments, however, these animals received a single injection of 20 mg/kg of memantine or vehicle 6 h after training, and then only vehicle following the 6 day retention interval. Both groups expressed no preference for the novel-located object in the test (mean + SEM exploration of the displaced object in the training: vehicle = 44.56 ± 3.82; memantine = 49.68 ± 3.21, and test: vehicle = 51.78 ± 1, 87; memantine = 56.39 ± 3.21; *t*(5) = −1.52; *p* = 0.18; *t*(5) = −1.89; *p* *=* 0.18; paired *t-*test; *n* = 6 per group). There were no differences between the groups (*t*(10) = 1.24; *p* = 0.24; unpaired *t*-test), indicating that LTM maintenance over time is not determined by early NMDAR blockade (data not shown).

In order to rule out the possibility of a non-specific memantine effect on forgetting, we performed the same experiment with another set of animals injecting every 12 h the potent NMDAR antagonist MK801. The MK801-treated group, but not the vehicle group, preferred exploring the displaced object in the test performed on day 7 compared with the training (*t*(8) = −9.29; *p* = 0.00001; *t*(5) = −0.98; *p* = 0.36, paired *t*-test; Fig. 2B). An additional set of animals kept receiving MK801 and was tested for memory retention on day 10. Interestingly, these animals were able to maintain the preference for the novel location object even at this time-point after training (*t*(6) = −5.52; *p* = 0.001, paired *t*-test), indicating that forgetting of spatial LTM is mediated through NMDAR-dependent signaling. Further analysis revealed that animals chronically treated with MK801 (for 7 or 10 d) significantly spent more time exploring the new object location than the control group (one-way ANOVA, *F*(2, 19) = 13.078; *p* = 0.0002, followed by SNK *post hoc* analysis, control group *vs* MK801 7 d, *p* = 0.0003, and control *vs* MK801 10 d, *p* = 0.03, respectively; Fig. 2C). Also, the groups did not differ in total exploration time (one-way ANOVA, *F*(2, 43) = 1.50; *p* = 0.23; Fig. 2D, middle panel), revealing that the treatment do not alter basal exploration activity.

Most of the studies regarding the mechanisms of forgetting (as well as the above results) were performed in hippocampal-dependent tasks^6^,^7^,^25^. We therefore next asked if the same mechanisms would be involved in a task that does not require the hippocampus, such as object recognition (OR) paradigm^26^. In this case, instead of moving an object to a new location during the test, one of the familiar objects was replaced with a new one and remained at its original location. Animals were trained in the OR task and tested one week later for memory retention (Fig. 2C). As in the above experiments, we injected systemically the NMDAR antagonist memantine (20 mg/kg) or its vehicle once a day between training and test. The control group expressed no preference for the novel object in the test (*t*(5) = 1, 07; *p* = 0.33, paired *t*-test), suggesting that object recognition memory was forgotten at this point in time. However, the memantine 20 mg/kg-treated group preferred exploring the new object to the old one presented in the training (*t*(7) = −3, 87; *p* = 0.006, paired *t*-test). Further, the group treated with memantine showed enhanced OR memory 7 d after training compared to the control group (*t*(12) = 2, 23; *p* = 0.04, unpaired *t*-test). As expected, the overall time spent exploring the objects was not different between the groups (*t*(26) = 1.14; *p* = 0.26, unpaired *t*-test; Fig. 2D, right panel). These data suggest that NMDAR activation is involved in forgetting of memories that are not maintained in the hippocampus^26^.

Taken together, these results provide additional support for the notion that forgetting requires NMDAR-mediated signaling.

### GluN2B-containing NMDAR regulates LTP decay in the hippocampus

NMDAR are heterotetramers. In the mammalian brain, most NMDARs are comprised of either GluN1 and GluN2A or GluN1 and GluN2B. Yashiro and Philpot^27^ have shown that, depending on the NMDAR subunit composition, its activation may induce distinct plasticity patterns, such as LTD or LTP^27^. The GluN2B-containing NMDAR enables a higher Ca^2+^ influx into postsynaptic neurons, and has been associated with plastic events such as fear memory destabilization^28^ and memory updating^23^,^24^. Recently it has been suggested that the GluN2B might be critical in regulating forgetting^4^,^5^. Indeed, strong emotional memories that are not forgotten have been associated with downregulation of GluN2B^10^.

**Figure 3:**
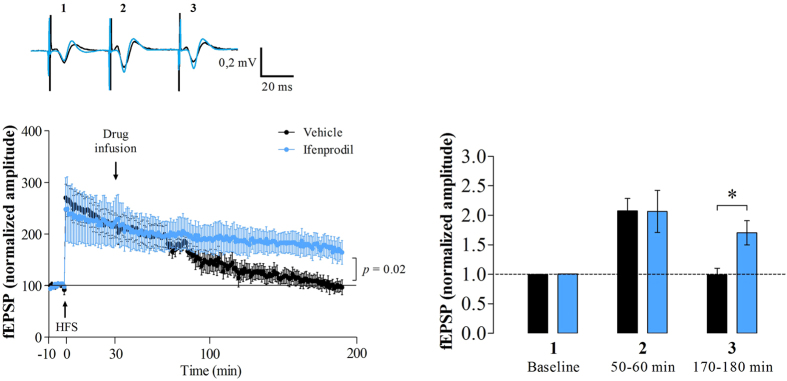
Hippocampal LTP decay *in vivo* is regulated by GluN2B-NMDAR. Corresponding representative traces of anesthetized rats infused with vehicle or ifenprodil (GluN2B-NMDAR antagonist) 30 min after LTP induction. The plot presents CA1-evoked synaptic potentials recorded for 180 min (left panel). In this protocol, hippocampal LTP in the control group start to decay around 90 min after HFS, whereas the application of the GluN2B-NMDAR antagonist prevents the natural decay of LTP. Right panel shown the% of change of normalized amplitude in three distinct intervals (1 = baseline response; 2 = 50–60 min after HFS; 3 = 170–180 min after HFS) (*n* = 4 per group).

To address whether GluN2B-NMDARs would be involved in the decay of hippocampal LTP, we used a protocol that induces LTP decaying within 90 min after the HFS^25^. In this case, thirty minutes after administering HFS in anesthetized animals, we infused the GluN2B-NMDAR antagonist ifenprodil or its vehicle in the CA1 region of the hippocampus. This protocol induced LTP in both the ifenprodil-treated group (206.6% ± 1.35 at 50 min after the HFS; *p* = 0.014 *vs* baseline; repeated measures ANOVA followed by SNK *post hoc* test) and in the control group (207.6% ± 1.36; *p* = 0.003 *vs* baseline; repeated measures ANOVA followed by SNK *post hoc*). Nonetheless, 180 min after the HFS, in the control group, LTP has decayed back to basal levels (99.2% ± 3.25; *p* = 0.972 *vs* baseline; repeated measures ANOVA followed by SNK *post hoc* test). However, intrahippocampal infusions of ifenprodil induced a nondecaying LTP (170.36% ± 4.20; *p* = 0.03 *vs* baseline; repeated measures ANOVA followed by SNK; Fig. 3). Further analysis revealed that the ifenprodil-treated group presented a higher amplitude 180 min after the HFS comparing with their relative controls (*t*(6) = 3.03; *p* = 0.02, independent *t*-test), but not 50 min after the HFS (*t*(6) = −0.02; *p* = 0.98, independent *t*-test). In addition, we performed the same experiment as described above using a lower ifenprodil concentration (0.1 μg/μL). No difference was found in the 10 min prior to HFS, *t*50–60 min or *t*170–180 min (*p* > 0.05, data not shown). An additional experiment was performed in order to test whether ifenprodil affects basal transmission in the hippocampus. No difference was found between ifenprodil and vehicle groups (data not shown).

Taken together, these data show that GluN2B-containing NMDAR is robustly engaged in the decay of short-lasting LTP in the hippocampus.

### Ca^2+^-dependent signaling is essential to regulate spatial memory maintenance: role of LVDCCs

**Figure 4:**
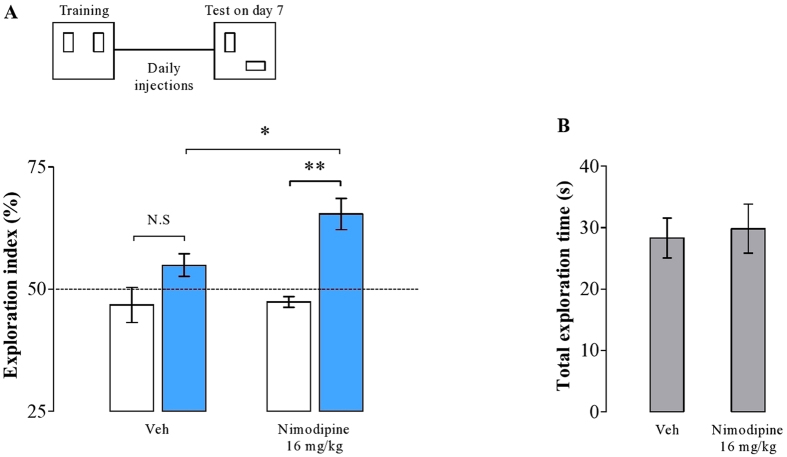
Ca2^+^ -dependent signaling through LVDCCs regulates long-term memory maintenance. (**A**) Rats treated chronically with nimodipine, a LVDCC blocker, significantly spent more time exploring the new object location than the control group during the test conducted 7 d after training. White bars represent the performance in the training, and blue bars in the test. (**B**) The overall exploration does not differ between the groups. Data expressed as mean ± SEM. *p < 0.5, **p < 0.01 (*n* = 6 per group).

The mechanisms that promote forgetting of LTM may engage various Ca^2+^ signaling pathways. For example, the L-type voltage dependent calcium channel (LVDCC) has been associated with memory destabilization required during memory updating^12^,^13^,^23^,^24^. To address whether LVDCC activation is also involved in forgetting, we trained animals in the OL paradigm, as described above. Animals were systemically treated with the LVDCC inhibitor nimodipine or its vehicle once per day, and tested 7 d after training. As shown in Fig. 4A, nimodipine-treated animals exhibited enhanced OL memory when tested 7 d after training (*t*(10) = 2, 65; *p* = 0.02, unpaired *t*-test). As expected, the control group expressed no preference for the novel-located object in the test (*t*(5) = −1.56; *p* = 0.17, paired *t*-test). However, rats treated with nimodipine preferred the displaced object to the object at the old location (*t*(5) = −4, 30; *p* = 0.007, paired *t*-test). Both groups did not differ in total exploration time (*t*(22) = −0.29; *p* = 0.77, unpaired *t*-test; Fig. 4B), suggesting that the treatment did not alter basal exploratory activity.

In conclusion, our results show for the first time that forgetting of long-term memory depends on the Ca^2+^ influx through LVDCC.

### Calcineurin positively modulates long-term memory forgetting

**Figure 5:**
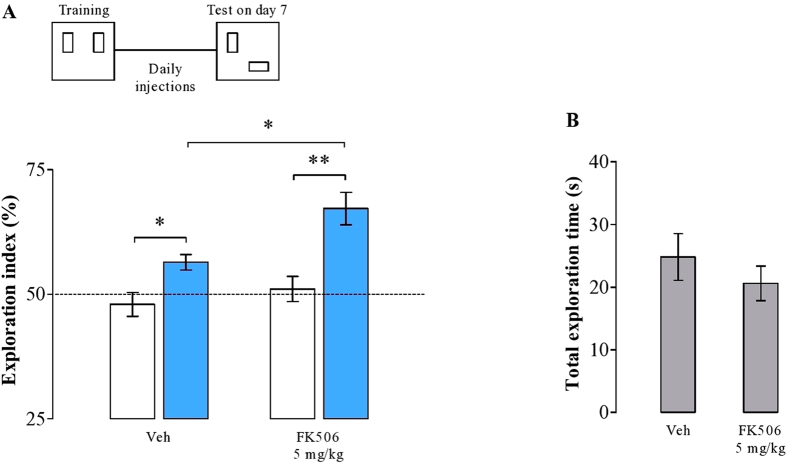
Calcineurin activation induces forgetting of the OL memory. (**A**) Compared to saline-treated group, rats treated with FK506, an inhibitor of the protein phosphatase calcineurin, significantly spent more time exploring the new object location during the drug-free retention test conducted 7 d after learning. White bars represent the performance in the training, and blue bars in the test. (**B**) The overall exploratory activity indicates no differences among groups. Data expressed as mean ± SEM. *p < 0.5, **p < 0.01 (*n* = 8–9 per group).

The above experiments have shown that the Ca^2+^ influx through both LVDCCs and NMDARs regulates forgetting of object location memory. Next, we investigated possible signaling pathways activated by the Ca^2+^ influx. Phosphatases are thought to negatively regulate synaptic plasticity and memory^15^,^16^,^17^,^18^,^19^. The phosphatase CaN is rapidly activated by Ca^2+^ influx^29^, requiring both NMDAR and LVDCC activation^30^,^31^. We predicted that if the Ca^2+^ influx activates CaN in order to induce forgetting, then inhibiting CaN activity would maintain LTM in a nondecaying state. To test this hypothesis, rats were trained in the OL task, chronically treated with the CaN inhibitor FK506 or their vehicle, and tested 7 d later. As shown in Fig. 5A, both groups preferred the displaced object to the object at the old location in the test (*t*(7) = −3, 33; *p* = 0.013 and *t*(8) = −3, 75; *p* = 0.005, paired *t*-test; vehicle and FK506, respectively). However, FK506-treated animals exhibited enhanced OL memory when tested one week after training (*t*(15) = 2, 86; *p* = 0.01, unpaired *t*-test). Both groups did not differ in total exploration time (*t*(32) = 0, 91; *p* = 0.36; unpaired *t*-test; Fig. 5B), suggesting that FK506 did not affect exploratory activity.

This outcome confirms that calcineurin plays an essential role in forgetting of long-term memory.

## Discussion

Here we explored neurobiological mechanisms underpinning the time-dependent forgetting of long-term memories. Based on theoretical models as well as experimental findings implicating NMDAR activation in forgetting^4^,^5^,^6^,^7^,^25^, we hypothesized a role for Ca^2+^ signaling in this form of memory loss. To this end, we blocked the activation of NMDARs, LVDCCs as well as calcineurin during the memory retention interval and found that these interventions prevented time-dependent forgetting, thus preserving LTM. Testing the role of NMDAR-dependent signaling in decay of LTP *in vivo*, we found that it required activation of GluN2B-containing NMDARs.

Forgetting has been shown to depend on NMDAR activation because long-term spatial memories acquired in the water maze as well as memories acquired in a radial arm maze can be maintained with chronic post-acquisition blockade of NMDAR^6^,^7^. Our results confirm these findings, as systemic injections of memantine and MK801 prevented the forgetting of long-term OL memory (Fig. 2A,B). In addition, we demonstrate that the natural forgetting of OR memory for object identity was also prevented with daily injections of memantine (Fig. 2C). In light of the finding that long-term object location, but not object identity memories are maintained in the hippocampus^26^, our results could indeed suggest that NMDAR-dependent forgetting occurs in various brain regions. The systemic administration of the NMDAR antagonists in our experiments, however, does not establish that hippocampal signaling is not involved in the forgetting of extra-hippocampal long-term memories. Future studies employing targeted interventions will be required to address this issue effectively.

In addition to NMDAR activation, we found that the loss of memory over time also requires Ca^2+^ influx through LVDCCs. Blocking their activation systemically with nimodipine was equally effective in maintaining long-term object location memories as was the administration of NMDAR antagonists (Fig. 4A). It appears therefore that neither NMDARs nor LDVCCs alone will provide a sufficient increase in Ca^2+^ levels in order to promote memory loss, but that activation of both will be required to trigger the relevant molecular mechanisms. These mechanisms will likely involve dephosphorylation of targets of the protein phosphatase calcineurin (CaN), because blocking its activity with chronic systemic injection of FK506 also sustained long-term memory (Fig. 5A). Interestingly, the reduction of AMPAR currents during LTD has been associated with CaN-dependent dephosphorylation of stargazin, an auxiliary protein that stabilizes AMPAR in the postsynaptic density of hippocampal neurons^21^. Calcineurin further promotes activity of protein phosphatase 1 (PP1), which has been implicated in the forgetting of long-term spatial memory^32^, suggesting a possible signaling pathway that leads to the loss of synaptic potentiation and memory.

Given these results, the most likely candidate processes that describe possible physiological mechanisms mediating this form of time-dependent forgetting of long-term memory is depotentiation, which leads to the reversal of LTP. Similar to the forgetting of object location memories, depotentiation requires the activation of both NMDARs and LDVCCs, and requires CaN-dependent signaling, unlike LTD, which is not affected by the CaN inhibitor FK506^17^,^33^. Specifically, depotentiation depends on GluN2B-containing NMDARs, as it can be blocked with ifenprodil and Ro25–6981, two selective inhibitors of GluN2B-NMDARs^34^. Our findings provide further support for a role of GluN2B-NMDAR activation in forgetting as we could prevent the natural decay of E-LTP in the hippocampus *in vivo* (Fig. 3).

Taken together, our findings provide support for a model that links Ca^2+^ -dependent signaling to a reduction of synaptic potentiation, which will likely involve the synaptic removal of postsynaptic GluA2-AMPARs. In fact, long-term memory maintenance as well as persistence of LTP requires the continuous stabilization of GluA2-containing AMPARs at postsynaptic sites^11^,^26^. Forgetting of aversive and spatial memories as well as hippocampal LTP decay also involves activity-dependent endocytosis of postsynaptic GluA2-AMPARs^25^. In this scenario, we suggest that the activity-dependent removal of postsynaptic GluA2-AMPARs may be initiated by NMDAR and LVDCC activation, which leads to CaN-dependent dephosphorylation of stargazin, thus affecting AMPAR trafficking. Nonetheless, since NMDAR are also present presynaptically, and contribute to LTP maintenance, we cannot determine to what extent presynaptic NMDARs mediate the effects described here.

Despite the fact that decay has been largely rejected as a significant source of forgetting of long-term memory, our present data suggest that a biological mechanism “actively” promotes the decay of established long-term memories as well as hippocampal LTP. Thus, forgetting likely represents a well-regulated brain-wide Ca^2+^ -dependent process that erases different types of memories. Whether memories are remembered or forgotten may thus not be a question of their actual strength but their ability to withstand forgetting. The natural duration of long-term object location memories that rats acquired in our training protocol was no more than five days. Yet, blocking NMDAR activation after learning with daily systemic injections of the uncompetitive NMDAR antagonist MK801 extended memory for 10 days, the longest time point we explored in our experiments (Fig. 2C).

In conclusion, there may be no principal limit to the potential duration of memories, as they may persist indefinitely for as long as they are protected from active forgetting. This perspective on memory loss may suggest alternative targets to approaches that seek to shorten or extend the duration of specific memories, and open a new window to the search of putative treatments for age-related cognitive decline.

## Additional Information

**How to cite this article**: Sachser, R. M. *et al.* Forgetting of long-term memory requires activation of NMDA receptors, L-type voltage-dependent Ca^2+^ channels, and calcineurin. *Sci. Rep.* **6**, 22771; doi: 10.1038/srep22771 (2016).
